# Capacitive Micro Pressure Sensor Integrated with a Ring Oscillator Circuit on Chip

**DOI:** 10.3390/s91210158

**Published:** 2009-12-14

**Authors:** Ching-Liang Dai, Po-Wei Lu, Chienliu Chang, Cheng-Yang Liu

**Affiliations:** 1 Department of Mechanical Engineering, National Chung Hsing University, Taichung, 402, Taiwan; E-Mail: player0912@hotmail.com; 2 Corporate R&D Headquarter, Canon Inc., Tokyo 146-8501, Japan; E-Mail: clchang6@ntu.edu.tw; 3 Center for Measurement Standards, Industrial Technology Research Institute, Hsinchu, 300, Taiwan; E-Mail: CY_Liu@itri.org.tw

**Keywords:** micro pressure sensors, ring oscillators, CMOS-MEMS

## Abstract

The study investigates a capacitive micro pressure sensor integrated with a ring oscillator circuit on a chip. The integrated capacitive pressure sensor is fabricated using the commercial CMOS (complementary metal oxide semiconductor) process and a post-process. The ring oscillator is employed to convert the capacitance of the pressure sensor into the frequency output. The pressure sensor consists of 16 sensing cells in parallel. Each sensing cell contains a top electrode and a lower electrode, and the top electrode is a sandwich membrane. The pressure sensor needs a post-CMOS process to release the membranes after completion of the CMOS process. The post-process uses etchants to etch the sacrificial layers, and to release the membranes. The advantages of the post-process include easy execution and low cost. Experimental results reveal that the pressure sensor has a high sensitivity of 7 Hz/Pa in the pressure range of 0–300 kPa.

## 1. Introduction

Microelectromechanical system (MEMS) technology has become popular for the miniaturization of sensors. The advantages of micro sensors fabricated by MEMS technology include small size, easy mass-production and low cost. Many micro capacitive pressure sensors have recently been manufactured by using MEMS technology. For instance, Habibi *et al.* [1] utilized a surface micromachining process to fabricate a capacitive pressure sensor array on a glass substrate. The array consisted of electrically parallel individual sensors with composite SiO_2_-Cr-SiO_2_ diaphragms and vacuum-sealed cavities underneath, and the cavities were formed by etching an aluminum sacrificial layer. Sippola and Ann [2] presented a ceramic capacitive pressure sensor fabricated by using thick film screen-printing technique, which the sensor consisted of a bottom electrode deposited on an alumina substrate and a top electrode deposited on a ceramic diaphragm. The cavity and diaphragm were created using a thick film sacrificial layer, and the pressure sensor had a sensitivity of 9.2 fF/psi. Wang and Ko [3] employed the silicon fusion bonding technique to develop a touch mode capacitive pressure sensor, and the sensor had a good linearity in the operating range and had a overload protection. A capacitive pressure sensor with a sandwich structure, proposed by Zhou *et al.* [4], was fabricated using a three-mask process and an anodic bonding, which the sensitivity of the sensor was 0.2 pF/kPa. These pressure sensors [1-4] did not have integrated circuits on a chip, so they needed to be coupled with circuits by packaging, leading to an increase in parasitic capacitance. Because the parasitic capacitance increased, the noise of capacitive sensors raised, resulting in lowering the performance of sensors. Integrating capacitive sensors with circuits on a chip helps to reduce the parasitic capacitance and packaging cost, so that the sensors have the benefits of low noise, high performance and small area. Thereby, in this work we developed a capacitive pressure sensor integrated with a sensing circuit on chip.

The CMOS-MEMS technique [5-9] that uses the commercial CMOS process to manufacture MEMS devices has the capability of integration micro devices with circuits on chip. In this study, we employ the CMOS-MEMS technique to fabricate a capacitive pressure sensor integrated with a ring oscillator circuit on a chip. The circuit is utilized to convert the capacitance variation of the pressure sensor into the frequency output. The signal of capacitance-to-frequency conversion in the sensor has a potential for applications in wireless communication. The pressure sensor needs a post-CMOS process to release the suspended membrane and seal the cavities. The post-process adopts wet etching to etch the sacrificial layers to release the suspended membrane, and then an LPCVD parylene is used to seal the cavities. The experimental results show that the capacitive pressure sensor has a sensitivity of 7 Hz/Pa in the pressure range of 0–300 kPa. Most of commercial micro pressure sensors are not a monolithic sensor chip, and the sensors usually use the hybrid approach to combine readout circuits. The hybrid approach leads to increase packaging cost and signal noise. In this work, the pressure sensor is a monolithic sensor chip, so it has the advantages of low packaging cost and small area.

## 2. Design of the Pressure Sensor

**Figure 1.**
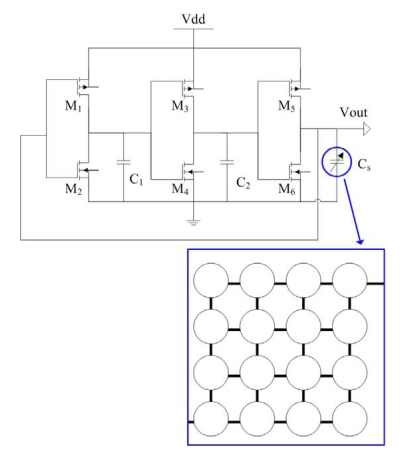
Schematic structure of the pressure sensor with ring oscillator.

Figure 1 shows the schematic structure of the capacitive pressure sensor with the ring oscillator circuit, where *C_s_* is the capacitive pressure sensor that is composed of 16 sensing cells in parallel. The pressure sensor changes in capacitance when applying a pressure to the sensing cells. The ring oscillator circuit is utilized to convert the capacitance variation in the pressure sensor into the frequency output.

**Figure 2.**
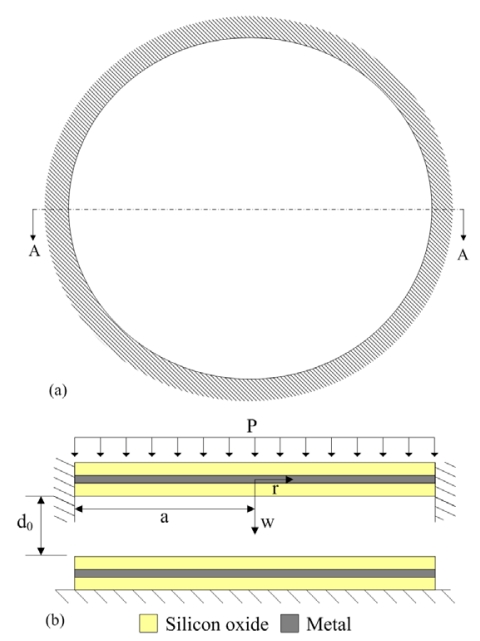
Schematic structure of a sensing cell, (a) top view; (b) AA cross-sectional view.

All sensing cells have the same structures and dimensions. Each sensing cell, as shown in Figure 2(a), is a circular shape of 100 μm diameter. Figure 2(b) illustrates the cross-section of the AA line of a sensing cell, which it is a parallel-plate capacitor. The upper electrode is a membrane, and it is a sandwiched structure consisting of a metal and two silicon dioxide layers. The lower electrode is a metal layer to be fixed on the silicon substrate. An air gap between the upper and lower electrodes is about 0.64 μm. The thickness of all silicon oxide layers is about 1 μm. As shown in Figure 2(b), supposing that a uniformly distributed pressure *p* acts on a clamped circular plate with radius *a*, the displacement equation of equilibrium of the plate is given by [10]:

(1) $$D\nabla^{2}\left(\nabla^{2}w(r)\right)=p$$

and:

(2) $$D=\frac{Eh^{3}}{12(1-\nu^{2})}$$

where *w*(*r*) represents the displacement of the plate; *E* is the Young's modulus of the plate; *h* is the thickness of the plate and *ν* is the Poisson's ratio. Solving Equation (1), the displacement of the clamped plate can be obtained [11]:

(3) $$w(r)=\frac{p}{64D}{\left(a^{2}-r^{2}\right)}^{2}$$

A sensing cell can be taken as a series of three capacitors and the total capacitance, *C_t_*, is given by:

(4) $$C_{t}=\frac{1}{\frac{1}{C_{\text{ox}}}+\frac{1}{C_{\text{gap}}}+\frac{1}{C_{\text{ox}}}}$$

and:

(5) $$C_{\text{ox}}={ɛ}_{\text{ox}}\frac{A}{d_{\text{ox}}}$$

(6) $$C_{\text{gap}}=\int_{0}^{2\pi }\int_{0}^{a}ɛ\frac{rdrd\theta }{d_{0}-w(r)}$$

where *C_ox_* and *C_gap_* represent the individual capacitance of the silicon oxide and air gap, respectively; *ε* is the permittivity of air; and *d*_0_ is the air gap at rest; *ε_ox_* is the permittivity of silicon oxide; *A* is the area of electrode plate; *d_ox_* is the thickness of silicon oxide. Substituting Equation (3) into Equation (6), the capacitance of air gap can be evaluated as:

(7) $$C_{\text{gap}}=\{\begin{array}{c} \frac{ɛ\pi a^{2}}{d_{\text{ox}}},\;p=0\\ 4ɛ\pi \sqrt{\frac{D}{d_{0}p}}\ln\left(\frac{\sqrt{d_{0}}+a^{2}\sqrt{\frac{p}{64D}}}{\sqrt{d_{0}}-a^{2}\sqrt{\frac{p}{64D}}}\right) \end{array},\;p>0$$

The capacitive pressure sensor is constructed by 16 sensing cells, so the capacitance, *C_s_*, of the pressure sensor can be expressed as:

(8) $$C_{s}=\frac{16}{\frac{1}{C_{\text{ox}}}+\frac{1}{C_{\text{gap}}}+\frac{1}{C_{\text{ox}}}}$$

**Figure 3.**
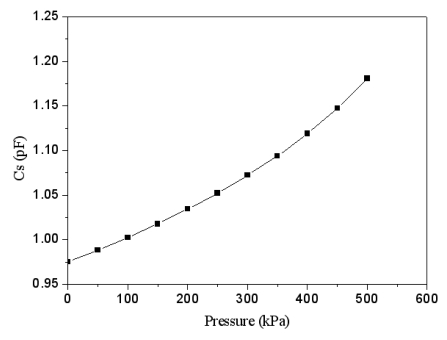
Relation between capacitance and pressure in the pressure sensor.

According to Equation (7), we know that the variation of capacitance *C_gap_* depends on the pressure *p*. In Equation (8), the capacitances *C_ox_* are constant, and the variation of capacitance *C_s_* relies on the capacitance *C_gap_*. Thus, the capacitance *C_s_* of the pressure sensor changes as the pressure *p* varies. In this design, the radius and thickness of the plate in the sensing cell are 50 μm and 2.6 μm, respectively. The material of the metal layers in Figure 2(b) is aluminum. The Young's moduli of aluminum and silicon oxide are 70 GPa and 69 GPa, respectively [12]. Thereby, suppose that the Young's modulus of the plate is 69.5 GPa, and the Poisson's ratio of the plate is 0.25. Substituting *E* = 69.5 GPa, *ν* = 0.25, *h* = 2.6 μm, *a* = 50 μm, *d_ox_* = 1 μm, *d*_0_ = 0.64 μm, *ε* = 8.85 × 10^−12^ F/m and *ε_ox_* = 3.54 × 10^−11^ F/m into Equations (2), (5), (7) and (8), the variation of capacitance *C_s_* in the pressure sensor related to the pressure *p* can be obtained, and the results are shown in Figure 3. The results reveal that the capacitance of the pressure sensor changes from 0.97 pF to 1.18 pF as the pressure increases from 0 to 500 kPa.

**Figure 4.**
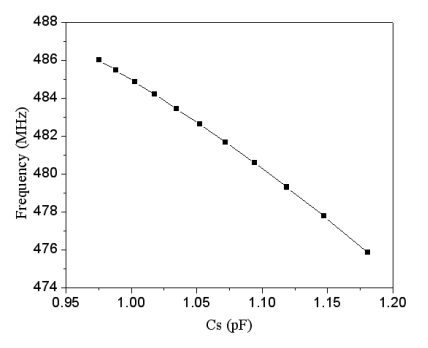
Oscillation frequency of the ring oscillator.

The capacitance variation of the pressure sensor is converted into the output voltage using the ring oscillator circuit. The professional circuit simulation software, HSPICE, is used to simulate the output signal of the ring oscillator circuit. As shown in Figure 1, *M_1_, M_3_* and *M_5_* are PMOS; *M_2_, M_4_* and *M_6_* are NMOS, where the capacitance of *C_1_* and *C_2_* is 0.5 pF. Figure 4 displays the simulated results of the ring oscillator. In this simulation, the input voltage *V_dd_* of 3.3 V is adopted. The simulated results depict that the oscillation frequency of the ring oscillator changes from 486 to 476 MHz as the capacitance of the pressure sensor increases from 0.97 to 1.18 PF.

**Figure 5.**
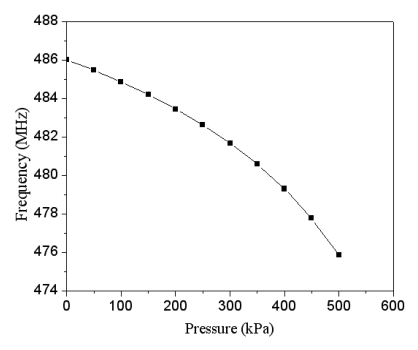
Simulated results of output frequency in the pressure sensor with ring oscillator.

**Figure 6.**
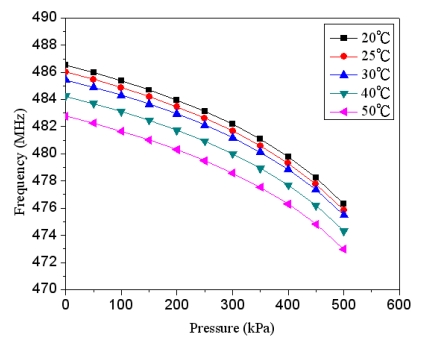
Output frequency of the pressure sensor at different temperatures.

Combining the data in Figures 3 and 4, we can obtain the relation between the output frequency and the pressure in the pressure sensor with ring oscillator circuit, and the results are plotted in Figure 5. The results present that the output frequency of the pressure sensor changes from 486 to 476 MHz as the pressure varies from 0 to 500 kPa. Furthermore, in order to characterize the effect of temperature, the output signal of the circuit with different temperatures is simulated using HSPICE. Figure 6 shows the output frequency of the pressure sensor at different temperatures. In this investigation, the input voltage *V_dd_* of 3.3 V is adopted. As shown in Figure 6, the output frequency drifts from 486 to 482.9 MHz at zero pressure when temperature increases from 25 to 50 °C, and the signal drifts from 746 to 743 MHz at the pressure of 500 kPa as temperature changes from 25 to 50 °C. The drift of frequency-to-temperature was about 0.16 MHz/°C, and the value was no better than that of Jornod and Rudolf [13].

## 3. Fabrication of the Pressure Sensor

**Figure 7.**
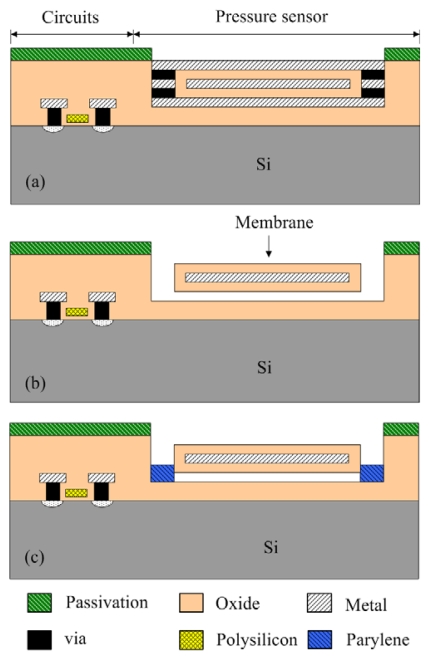
Process flow of the pressure sensor: (a) after completion of CMOS process, (b) etching the sacrificial layers, and (c) sealing the etch holes.

The commercial 0.35 μm CMOS process of Taiwan Semiconductor Manufacturing Company (TSMC) is employed to fabricate the capacitive pressure sensor. Figure 7 illustrates the fabrication flow of the capacitive pressure sensor. Figure 7(a) shows the schematic cross-section of the capacitive pressure sensor after completion of the CMOS process. In order to release the suspended membranes, the pressure sensor needs a post-CMOS process to remove the sacrificial layers [14]. The membranes are composed of the oxide, metal and oxide layers. The sacrificial layers are the metal and via layers, which the materials of the metal and via layers are aluminum and tungsten, respectively. In the post-process, the wet etching is used to etch the sacrificial layers, and to release the suspended membranes.

**Figure 8.**
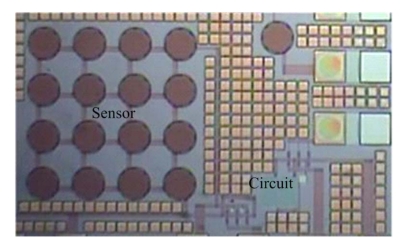
Photograph of the pressure sensor after the wet etching process.

**Figure 9.**
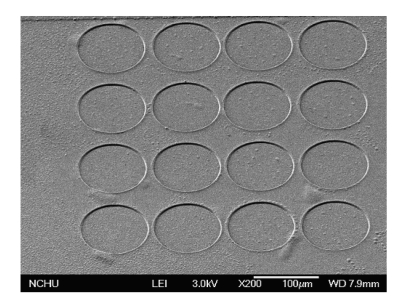
SEM image of the pressure sensor after depositing the parylene film.

Figure 7(b) displays that the pressure sensor is immersed in two etchants: one is a tungsten etchant with sulfuric acid and hydrogen peroxide in the ratio 2:1 and the other is an aluminum etchant with phosphoric acid, nitric acid, acetic acid and DI water in the ratio 14:1:2:3. An air gap between the membrane and substrate is formed after the sacrificial layers are removed. Figure 8 presents the photograph of the capacitive pressure sensor with ring oscillator circuit after the wet etching process. The etch holes in the pressure sensor have to be sealed. Figure 7(c) displays that the etch holes are sealed by using a LPCVD parylene, and then the parylene film is patterned by a dry etching. Figure 9 shows a scanning electron microscopy (SEM) image of the pressure sensor after depositing the parylene film. The parylene film has a thickness of about 2 μm. The pressure sensor is an absolute pressure sensor because the etch holes are sealed by LPCVD parylene in a high vacuum chamber.

## 4. Results and Discussion

A pressure chamber, a power supply and a spectrum analyzer were adopted to measure the characteristic of the capacitive pressure sensor with ring oscillator circuit. The pressure sensor was mounted in the pressure chamber, and a calibrated pressure sensor was utilized to monitor the gas pressure in the pressure chamber. The nitrogen pressure source was provided to the pressure chamber, and the nitrogen pressure in the chamber could be tuned by the gas valves. The power supply provided the *V_dd_* voltage of 3.3 V to the ring oscillator circuit. The frequency response of the pressure sensor was detected using the spectrum analyzer.

**Figure 10.**
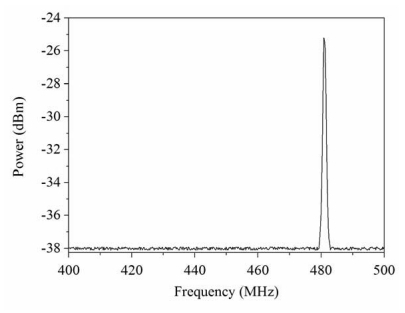
Frequency response of the pressure sensor at zero pressure.

**Figure 11.**
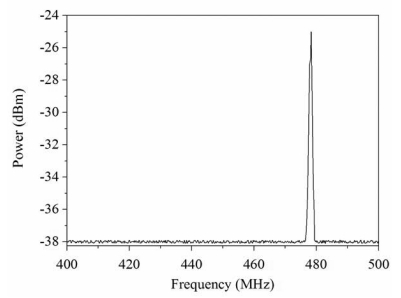
Frequency response of the pressure sensor at 400 kPa pressure.

**Figure 12.**
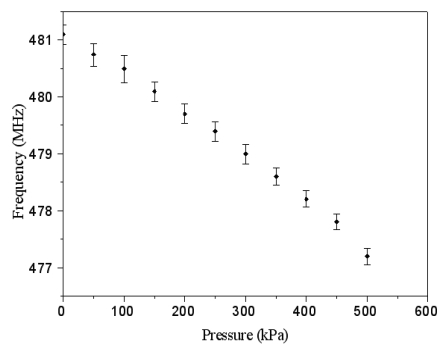
Measured results of output frequency in the pressure sensor with ring oscillator.

Figure 10 displays the measured results of frequency response for the pressure sensor at zero pressure. The results showed that the pressure sensor had an output frequency of 481.1 MHz at zero pressure. When increasing the nitrogen pressure in the chamber, the output frequency of the pressure sensor produced a change. Figure 11 presents the frequency response of the pressure sensor at the pressure of 400 kPa. The measured results depicted that the output frequency of the pressure sensor changed to 478.1 MHz at 400 kPa pressure. Figure 12 shows the output frequency of the pressure sensor at room temperature and different pressures. The curve in Figure 12 was a linear in the range of 0–300 kPa, and the output frequency changed from 481.1 to 479 MHz when the pressure increased from 0 to 300 kPa. Thus, the sensitivity of the pressure sensor was about 7 Hz/Pa in the range of 0–300 kPa. Comparing with the simulated results in Figure 5, the measured results of the output frequency were close to the simulated results.

A capacitive pressure sensor integrated with the readout circuit, reported by Dai and Liu [15], was fabricated using the CMOS-MEMS technique, and the pressure sensor consisted of 128 sensing cells. Each sensing cell was a hexagon that was tangential to the periphery of a circle with 100 μm diameter. In this work, the pressure sensor was composed of 16 sensing cells and each sensing cell was a circle with 100 μm diameter, so the area of the pressure sensor was less than that of Dai and Liu [15]. On the other hand, Dai and Liu [15] used the readout circuit to convert the capacitance of the pressure sensor into the voltage output. This work employed the ring oscillator circuit convert the capacitance of the pressure sensor into the frequency output, which was different from Dai and Liu [15], and the output signal of the sensor had a potential for applications in wireless transmission.

The sensitivity and area in many micro pressure sensors were summary in Table 1. Kudoh *et al.* [16] presented a capacitive pressure sensor integrated with the capacitance-to-frequency conversion circuits on chip manufactured by the CMOS process. The post-process of bulk micromachining and glass-to-silicon anodic bonding was adopted to achieve the monolithic sensor. The area of the senor chip was 3.3 × 3.7 mm^2^, and the sensitivity was about 2.86 Hz/mmHg. Liu *et al.* [17] employed the CMOS-MEMS technique to fabricate a monolithic capacitive pressure sensor with readout circuits. The post-process of the sensor adopted back-side etching silicon substrate to form a membrane and used glass-to-silicon anodic bonding to seal the cavity. The sensor structure was 800 × 800 μm^2^ in size, and the sensitivity of the sensor was 3.2 Hz/hPa. Chang and Allen [18] used a hybrid approach to combine the capacitive pressure sensor with a commercially available IC MS3110 (from Microsensors Inc.) on stainless steel substrate, and the sensors had a sensitivity of 44.8 mHz/kPa. Crescini *et al.* [19] manufactured a capacitive pressure sensor with a special electrode shape for improving linearity using a thick-film technology, and the sensor coupled with a relaxation oscillator circuit, which its sensitivity was 0.012 Hz/Pa. A comparison of the above literature, the sensitivity of the senor in this work exceeds that of Kudoh *et al.* [16], Liu *et al.* [17], Chang and Allen [18] and Crescini *et al.* [19]. The post-process in this study is easier than that of Kudoh *et al.* [16] and Liu *et al.* [17]. The sensor chip area of 600 × 800 μm^2^ in this work is smaller than that of Kudoh *et al.* [16].

## 5. Conclusions

The capacitive pressure sensor integrated with the ring oscillator circuit on chip has successfully been implemented using the commercial CMOS process and a post-process. The post-process used the etchants to etch the sacrificial layers to release the membranes of the pressure sensor, and then the etch holes in the pressure sensor were sealed by the LPCVD parylene. The advantages of the post-process were easy execution and compatible with the commercial CMOS process. The pressure sensor produced a change in capacitance upon applying a pressure to the sensor. The ring oscillator converted the capacitance variation of the pressure sensor into the frequency output. The experiments depicted that the pressure sensor had a sensitivity of about 7 Hz/Pa in the 0–300 kPa pressure range.

## Figures and Tables

**Table 1.** The sensitivity and area in many micro pressure sensors.

**Types**	**Sensitivity**	**Area**
Kudoh *et al.* [16]	2.86 Hz/mmHg	3.3 × 3.7 mm^2^
Liu *et al.* [17]	3.2 Hz/hPa	800 × 800 μm^2^
Chang and Allen [18]	44.8 mHz/kPa	
Crescini *et al.* [19]	0.012 Hz/Pa	
This work	7 Hz/Pa	600 × 800 μm^2^
